# Flexible Screen-Printed Gold Electrode Array on Polyimide/PET for Nickel(II) Electrochemistry and Sensing

**DOI:** 10.3390/s25133959

**Published:** 2025-06-25

**Authors:** Norica Godja, Saied Assadollahi, Melanie Hütter, Pooyan Mehrabi, Narges Khajehmeymandi, Thomas Schalkhammer, Florentina-Daniela Munteanu

**Affiliations:** 1 Interdisciplinary School of Doctoral Studies, “Aurel Vlaicu” University of Arad, 2-4 Elena Drăgoi Str., 310330 Arad, Romania; godja@attophotonics.com; 2 Attophotonics Bioscieneces GmbH, Viktor Kaplan Str. 2E, 2700 Wiener Neustadt, Austria; assadollahi@attophotonics.com (S.A.); melanie@attophotonics.com (M.H.); mehrabi@attophotonics.com (P.M.); khajeh@attophotonics.com (N.K.); t.sc@rhp.at (T.S.); 3 Faculty of Food Engineering, Tourism and Environmental Protection, “Aurel Vlaicu” University of Arad, 2-4 Elena Drăgoi Str., 310330 Arad, Romania

**Keywords:** screen-printed electrode array, 16 Au working electrodes, Ni^2+^ electrochemistry, water-based electrolyte systems, deep-eutectic solvents, simultaneous characterization of multiple analytes

## Abstract

Nickel’s durability and catalytic properties make it essential in the aerospace, automotive, electronics, and fuel cell technology industries. Wastewater analysis typically relies on sensitive but costly techniques such as ICP-MS, AAS, and ICP-AES, which require complex equipment and are unsuitable for on-site testing. This study introduces a novel screen-printed electrode array with 16 chemically and, optionally, electrochemically coated Au electrodes. Its electrochemical response to Ni^2+^ was tested using Na_2_SO_3_ and ChCl-EG deep eutectic solvents as electrolytes. Ni^2+^ solutions were prepared from NiCl_2_·6H_2_O, NiSO_4_·6H_2_O, and dry NiCl_2_. In Na_2_SO_3_, the linear detection ranges were 20–196 mM for NiCl_2_·6H_2_O and 89–329 mM for NiSO_4_·6H_2_O. High Ni^2+^ concentrations (10–500 mM) were used to simulate industrial conditions. Two linear ranges were observed, likely due to differences in electrochemical behaviour between NiCl_2_·6H_2_O and NiSO_4_·6H_2_O, despite the identical Na_2_SO_3_ electrolyte. Anion effects (Cl^−^ vs. SO_4_^2−^) may influence response via complexation or ion pairing. In ChCl-EG, a linear range of 0.5–10 mM (R^2^ = 0.9995) and a detection limit of 1.6 µM were achieved. With a small electrolyte volume (100–200 µL), nickel detection in the nanomole range is possible. A key advantage is the array’s ability to analyze multiple analytes simultaneously via customizable electrode configurations. Future research will focus on nickel detection in industrial wastewater and its potential in the multiplexed analysis of toxic metals. The array also holds promise for medical diagnostics and food safety applications using thiol/Au-based capture molecules.

## 1. Introduction

Nickel is a critical material across diverse industries due to its exceptional durability, corrosion resistance, and catalytic properties. It plays a vital role in aerospace, automotive, electronics, and fuel cell technologies. When alloyed with Au, Ni enhances electrical conductivity and mechanical stability in electronic components such as connectors and circuit boards. Moreover, Au-Ni hydroxides have shown great potential in energy storage systems like pseudocapacitors, offering high energy density and long-term performance [1]. Nickel’s widespread use in electroless and electrochemical deposition processes stems from its hardness and resistance to environmental degradation [2,3]. It also contributes to the protective properties of anodic sealing layers [4] and serves as a key catalyst in hydrogen and oxygen evolution reactions (HERs and OERs), which are central to fuel cell and water-splitting technologies [5]. Despite these advantages, nickel leaching during prolonged operation—particularly in aggressive conditions—can contaminate water sources and raise ecological and health concerns [6,7,8].

Regulatory agencies such as the USEPA and WHO have established strict concentration limits for Ni in water, which are set at 0.07 mg/L for drinking water and 0.1 mg/L for wastewater [9]. Traditional analytical techniques for nickel detection, including ICP-MS, AAS, and ICP-AES, offer excellent sensitivity, but are limited by their high cost, instrumentation complexity, and lack of portability. As such, they are unsuitable for routine or in situ applications. Electrochemical sensors have emerged as effective alternatives, offering portability, cost-efficiency, and ultra-trace sensitivity.

Voltammetry, particularly with advanced electrode-modification strategies, has proven to be a versatile and sensitive approach [10]. Materials such as nanostructured metals, conductive polymers, and carbon nanocomposites have significantly improved detection performance. Bimetallic nanocomposite sensors incorporating AgNPs on magnetite/DMG-modified carbon paste electrodes demonstrated a detection limit of 0.6 nM in river water [11]. Lead-film screen-printed electrodes (PbF-SPEs), prepared in situ in ammonia buffer, enabled simultaneous detection of Ni(II) and Co(II) with detection limits of 0.2 and 0.3 µg/L and relative standard deviations below 4% [12]. A Ni^2+^-imprinted polymer sensor based on Fe_3_O_4_@MWCNTs-modified CPE offered strong selectivity and a 0.27 µg/L detection limit across a wide linear range [13]. Renewable bismuth bulk annular band electrodes (RBiABEs) served as sustainable alternatives to film-based sensors, achieving 0.29 mg/L and 0.018 mg/L for Ni(II) and Co(II), respectively [14]. Detection using screen-printed carbon electrodes (SPCEs) with DPAdSV and DMG complexation allowed for mercury- and bismuth-free sensing, reaching a 0.5 µg/L limit and showing excellent reproducibility in certified wastewater samples [15]. MnO–chitosan composites on Pt electrodes enabled fast, simultaneous detection of Ni(II) and Se(IV), with a 0.718 µg/L detection limit and an 18 s response time [16]. MWCNT–BiOCl nanosheets, using the technique of chrono deposition combined with DPV stripping, enabled Ni^2+^ detection in the 20–160 µM range with a detection limit of 0.019 µM [17]. Integrating Nafion–graphene with DMG-modified glassy carbon electrodes enhanced stability and selectivity, achieving a 1.5 µg/L LOD in tap water with low interference from Zn^2+^ and Co^2+^ [18]. Carbon-based screen-printed electrodes (SPEs) have shown solid performance when coupled with adsorptive stripping voltammetry (AdSV), enabling quantification over a broad range of 7.6 to 200 g/L with a 2.3 µg/L LOD [19]. Incorporating bismuth–graphene composites on SPEs has further enhanced their electrochemical response under square-wave adsorptive stripping (SWAdS) conditions, achieving a 2.5 g/L LOD [20]. Nanostructured hybrids such as silver-modified bismuth oxybromide (Ag/BiOBr) have been employed using cyclic voltammetry (CV), achieving low micromolar detection limits (0.122 µM) and exhibiting extended linearity in the 10–40 µg/L range [21]. Electrodes based on bismuth biomaterials, assessed via CV and linear sweep voltammetry (LSV), enable detection over a broad concentration range (0.477–1.669 µg/L) and are regarded as environmentally friendly alternatives [22]. For improved selectivity, gold SPEs modified with calixarene and 3-mercaptopropionic acid have been employed in combination with differential pulse voltammetry (DPV), demonstrating suitability for Ni(II)/Zn(II) detection at higher concentration ranges (340 µg/L) [23]. Though typically used for high-concentration applications, platinum-based systems have achieved detection in the millimolar range (15–40 mM) using CV [24]. Colourimetric sensors based on sulfamethoxazole-quinolinol (SMAMQ) have achieved 40.1 nM detection with reusability [25].

Electrodeposition studies using NiCl_2_·6H_2_O in ethylene glycol showed high cathodic efficiency (88%) in chloride-free media, decreasing to 26% upon NaCl addition due to hydrogen evolution [26]. On Ag/GDC scaffolds, pulse plating techniques produced uniform nickel layers with improved coverage and reduced hydrogen pitting, relevant for solid oxide fuel cell applications [27]. Passivation behaviour in acidic environments results in Ni(OH)_2_ layer formation [28], with the NiOH^+^ intermediate considered essential in the reduction mechanism, as proposed by Bockris et al. [29]. The electrochemical reduction of Ni-bipyridine complexes revealed Ni(I) intermediates through comproportionation, with CV indicating three reversible redox waves [30]. Green chemistry approaches have introduced deep eutectic solvents (DESs), such as choline chloride ethylene glycol (ChCl–EG), which enable the formation of nanocrystalline Ni coatings with hardness improvements exceeding 100 HV over conventional baths [31]. Alcohol-based solvents (methanol, ethylene glycol, glycerol) have also been explored. Methanol enabled compact crystalline deposits, while ethanol and glycerol favoured salt-film formation due to Ni–alcoholate interactions [32].

Gold electrodes remain widely used due to their conductivity, stability, and electrochemical compatibility. Studies by Puglia et al. [33] showed that alloy composition strongly influences electrochemical behaviour, with pure gold displaying asymmetric CVs and no hydrogen adsorption in PBS. Screen-printed gold electrodes achieved Pb^2+^ detection at 0.5 µg/L using SWASV in a three-electrode configuration [34]. Further sensitivity improvements were realized through colloidal gold nanoelectrode ensembles (GNEEs), detecting As(III) and Hg(II) at ultra-trace levels (0.02 ppb) [35]. The modification of SPCEs with AuNPs enabled Hg^2+^ detection at 0.33 µg/L within a linear range of 0.5–80 µg/L and with a 5 min analysis time [36]. Gold electrodes have also been adapted for the detection of pharmaceuticals and pesticides, including thioridazine [37] and glyphosate. SAM functionalisation with cysteamine and copper significantly enhanced glyphosate detection on screen-printed gold electrodes [38,39].

Mechanistic studies of nickel deposition on gold electrodes have revealed that Ni(OH)_2_ forms below −0.5 V and nickel nucleation begins around −0.8 V in NiSO_4_ solutions [40]. Using ferrioxalate in citric acid as a redox mediator, the Ni(II) response improved via diffusion-controlled mechanisms [41]. In molten-salt systems (KNO_3_–KCl), potential-dependent anionic surface species were identified on gold electrodes [42].

DES media have shown promise in improving nickel electrodeposition by widening the electrochemical window and suppressing hydrogen evolution. Successful deposition of Ni, Ni-Co, Cu, Au, and Bi in DES formulations has been reported [43,44,45,46,47]. Simultaneous Ni–Co codeposition from ChCl–EG media enabled the formation of nanocrystalline alloys (3.1–4.0 nm), with their corrosion resistance depending on the cobalt content. Electrochemical analysis confirmed irreversible, diffusion-controlled behaviour with instantaneous 3D nucleation [45]. Nicholson [48] conducted early ASV studies with Au electrodes, showing reliable Ni(II) detection between 10^−5^ and 5 × 10^−8^ M in deoxygenated media.

To reduce gas evolution during deposition, Srivastava and Tikoo [49] employed NiCl_2_ in N,N-dimethylformamide (DMF) with HCl additives, achieving a cathodic efficiency of nearly 100%. Cui et al. [50] compared choline chloride–ethylene glycol (ChCl–EG) deep eutectic solvent and pure ethylene glycol (EG) systems, finding a diffusion-controlled, irreversible reduction in both media.

A novel platform of 16 screen-printed Au electrodes on flexible polyethylene terephthalate (PET) and polyimide (PI) substrates has been developed in this context. These sensors are suitable for lightweight, portable analysis and have been tested for Ni(II) detection. Their versatility makes them promising candidates for broader environmental monitoring.

## 2. Materials and Methods

### 2.1. Chemicals

Several commercial compounds and analytical reagents were used to construct the 16-electrode gold array and test its application in Ni^2+^ electrochemistry. Silver paste (Silverpaste 200-05, Dico Electronic, Schwabach, Germany >84% Ag, 0.010 square/mil), Ag/AgCl paste (Silver chloride paste 60:40, C2130809DS, Sunchemical/Gwent Electronic Materials, Jersey City, NJ, USA), and Thinner 112-19 (DICO Electronic) were employed for the conductive layers. For gold chemical deposition, we utilized sodium tetrachloroaurate(III) dihydrate (99%, Sigma Aldrich, Waltham, MA, USA, 298174), sodium sulphite anhydrous (*purum* p.a. >97%, Carl Roth, Karlsruhe, Germany), sodium ascorbate (L-ascorbic acid sodium salt, 99%, S3 Chemicals), ammonium chloride (Carl Roth), hydrochloric acid (25%, Sigma Aldrich), and Tween 20 (Carl Roth). Additionally, a white insulation layer (D2171220D2: White Dielectric Paste, BG04, Sunchemical/Gwent Electronic Materials) and a blue dielectric layer (D50706P3: Blue Dielectric Ink, BG04, Sunchemical/Gwent Electronic Materials) were evaluated. The electrochemical in situ gold deposition was tested using an 18-karat gold plating bath (TIFOO).

We developed screen-printed electrodes featuring silver substrates coated with gold, with the sensor design provided by Attophotonics Biosciences GmbH, Neustadt, Austria. The sensor system comprises 16 working electrodes, each with an area of 1.7 mm^2^, and a counter electrode made of gold-coated silver. The system is optimized to operate with a maximum analyte volume of 200 µL/electrode.

For electrochemical characterization, the following chemicals were utilized: ferricyanide [Fe(CN)_6_]^3–/4–^ in 0.1 M KCl, 0.1 M Na_2_SO_3_, 100 mM choline chloride in ethylene glycol (ChCl-EG), NiCl_2_, NiCl_2_ 6 H_2_O, and NiSO_4_ 6H_2_O.

### 2.2. Methods

The potentiostat developed in collaboration with the University of Vienna (input amplifier OPA 128, input bias 700 fA, current/voltage amplifier OPA 111), equipped with ZEUS NT9software, was employed for electrochemical measurements using the screen-printed gold (Au) electrodes, fabricated as detailed in Section 2.3. A ZEISS FE-SEM “ΣIGMA HD VP” equipped with a TEAM Pegasus EDX system and a TEAM Octane Plus SSD detector was used for qualitative surface analysis.

### 2.3. Fabrication of Screen-Printed Gold Sensors

**Figure 1:**
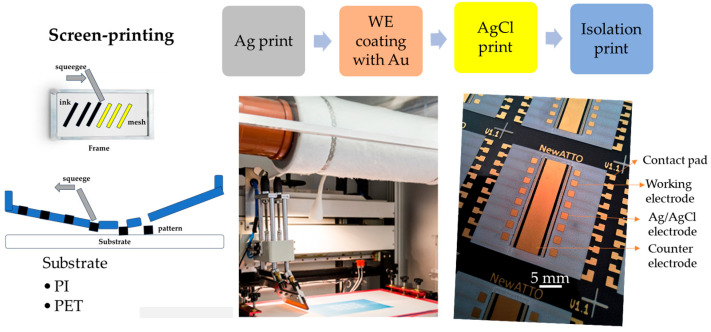
Step-by-step sensor fabrication: the process begins with the design of the electrodes; in this case, there are 16 working electrodes. Screen printing is employed to create a stencil for the electrode pattern. This is followed by preparing and precisely applying the electrode paste onto a polyimide (PI) or PET substrate, ensuring uniformity and adherence.

Using a gold electrode for nickel detection offers significant advantages due to gold’s stability and superior electrochemical properties. Gold is chemically inert and highly resistant to corrosion in both acidic and alkaline conditions, making it suitable for diverse electrochemical environments. Furthermore, its excellent conductivity ensures efficient electron transfer, enhancing electrochemical sensors’ sensitivity and overall performance. The screen printing technique was employed to produce the gold electrodes, with the step-by-step fabrication process schematically illustrated in Figure 1. This process involves sequential printing steps: first, silver (Ag) deposition, followed by gold (Au) deposition, the printing of the Ag/AgCl reference electrode, and, finally, the insulation layer. Subsequently, the working electrodes can be further modified by incorporating additional materials through electrodeposition or other chemical methods to enhance their performance. A microfluidic system is then integrated to manage fluid flow around the electrodes, and the entire system is prepared for sensor testing.

After screen printing, the silver layer was dried in an oven at 85 °C for 30 min. Subsequently, the sensors were subjected to a surface activation step by immersion in a 1% sodium hypophosphite (NaH_2_PO_2_) solution at 65 °C for 30 min. This pre-treatment was essential to prepare the surface for subsequent electroless gold deposition. The electroless gold (Au) plating process significantly improved the deposition efficiency, depositing an average of 45 mg of gold per sheet.

Ag/AgCl Reference Electrode: To ensure optimal consistency and performance, the Ag/AgCl paste was thoroughly mixed prior to use—ideally using an Ultra-Turrax homogeniser for a few minutes. The Ag/AgCl layer was dried at 85 °C for 30 min in an oven.

Dielectric Layer Application: Several measures were implemented to ensure a uniform and reliable dielectric layer:

- The white dielectric paste was diluted by 5% using Dico Electronics 112-19 thinner.
- The printer spacing was adjusted to enhance layer homogeneity.
- A multi-layer approach was used, filling the screen four times in a single print cycle to achieve uniform coverage.

These modifications led to consistent and repeatable results across multiple sensor batches.

Additional process parameters included the following:

- Screen design specifications: a Monolen 150-31W/Y sieve with a mesh size of 32 µm, a screen thickness of 47 µm ± 2 µm, and a thread diameter of 31 µm.

Figure 1 outlines the fabrication process, from printing to final curing, and clarifies the layer sequence, contact pads, and flexible substrate.

**Figure 2:**
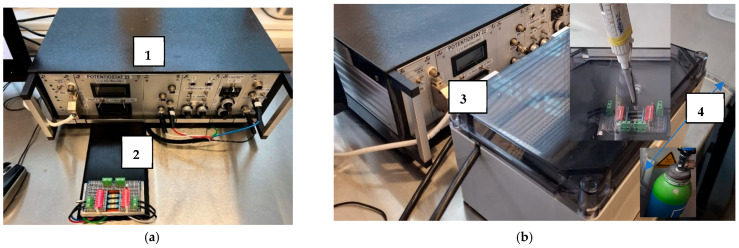
The measurement setup using a potentiostat (1) developed in collaboration with the University of Vienna and equipped with ZEUS software. (**a**) Measurements were made at room temperature in air. The electrode array (2) is connected to the potentiostat; (**b**) a box (3) was used for measurements at room temperature under argon gas (4). The blue arrow indicates the tube through which nitrogen flows into the box.

Figure 2 shows the experimental setup used for electrochemical measurements with this study’s newly developed electrode array. The system includes a custom-built potentiostat—developed in collaboration with the University of Vienna and operated using ZEUS software—and supports measurements in ambient air and under an argon atmosphere, enabling versatile testing conditions.

**Figure 3:**
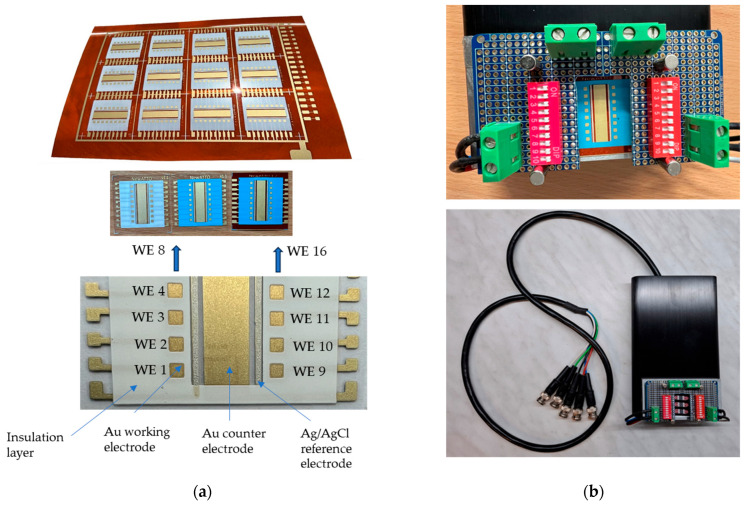
Sensors (12 sensors on a PI sheet) and integration: (**a**) Screen-printed sensors with a Au-sensor design, consisting of 16 working electrodes, a Au counter electrode, and a Ag/AgCl reference electrode, all printed on PI or PET substrates. Two insulation layers (white and blue) were tested. (**b**) The test setup in which the 16-electrode array was mounted for electrochemical measurements.

Twelve sensors were printed onto a PI sheet, each comprising 16 working electrodes, a common counter electrode, and a reference electrode (Figure 3a). For electrochemical measurements, a test setup was fabricated (Figure 3b) and integrated into the measurement box (see Figure 2b).

**Figure 4:**
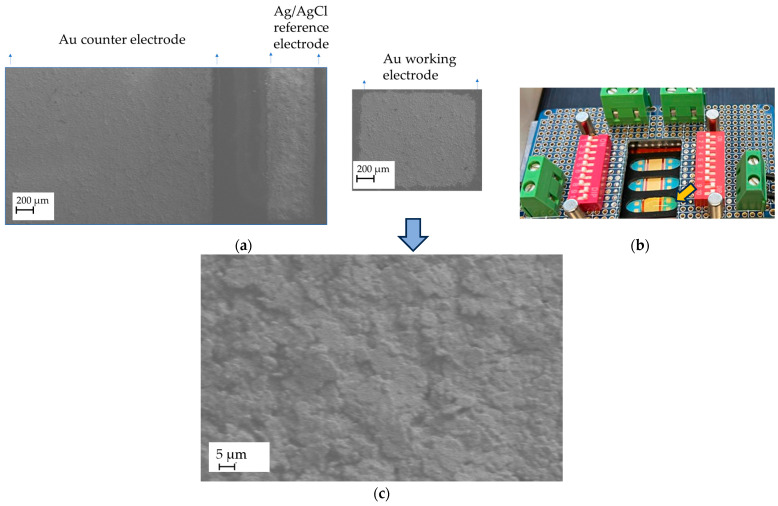
Surface morphology and electrolyte wetting of the working electrode with analyte: (**a**) An SEM image of the gold sensor, showing the gold working electrodes (WEs), gold counter electrodes, and Ag/AgCl reference electrodes screen-printed on the PI substrate; (**b**) Wetting of the working electrode with a nickel-containing supporting electrolyte (100–200 µL), indicated by the yellow arrow. The small volume of electrolyte resulted in a very low nickel concentration at the electrode surface, potentially reaching the nanomolar range. (**c**) A higher-magnification image showing the microstructured gold surface of the working electrode.

The quality of the screen-printed sensors was evaluated using Scanning Electron Microscopy (SEM) and Energy-Dispersive X-ray Spectroscopy (EDX). The area of each working electrode was measured and determined to be 1.7 mm^2^ (Figure 4a). The electrolyte was applied during electrochemical measurements to cover the working electrodes, the reference electrode, and the counter electrode, as illustrated in Figure 4b.

## 3. Results and Discussion

### 3.1. Electrochemical Measurements

Cyclic voltammetry (CV) is a powerful electrochemical technique that provides real-time, highly sensitive data on redox reactions. This is particularly useful for detecting nickel in deep eutectic media, where traditional analytical techniques might be less effective due to the complex nature of the electrolyte. Gold electrodes behave similarly to platinum, but have limited usefulness in the positive potential range above 800 mV due to oxidation of their surfaces. The Ni^2+^ electrochemistry on the screen-printed gold electrode array was studied using chronoamperometric cycles in Na_2_SO_3_ as the supporting electrolyte and cyclic voltammetry (CV) in ChCl-EG as the supporting electrolyte.

### 3.2. Analysis of Gold Electrodes

The electrochemical behaviour of the modified Au electrodes, specifically related to their active surface area (1.7 mm^2^), was evaluated using cyclic voltammetry. The redox response of the [Fe(CN)_6_]^3−^/^4−^ couple in 0.1 M KCl was examined to assess the quality of the Au coating. This system is a well-established probe for determining electrode surface integrity and electron-transfer kinetics.

**Figure 5:**
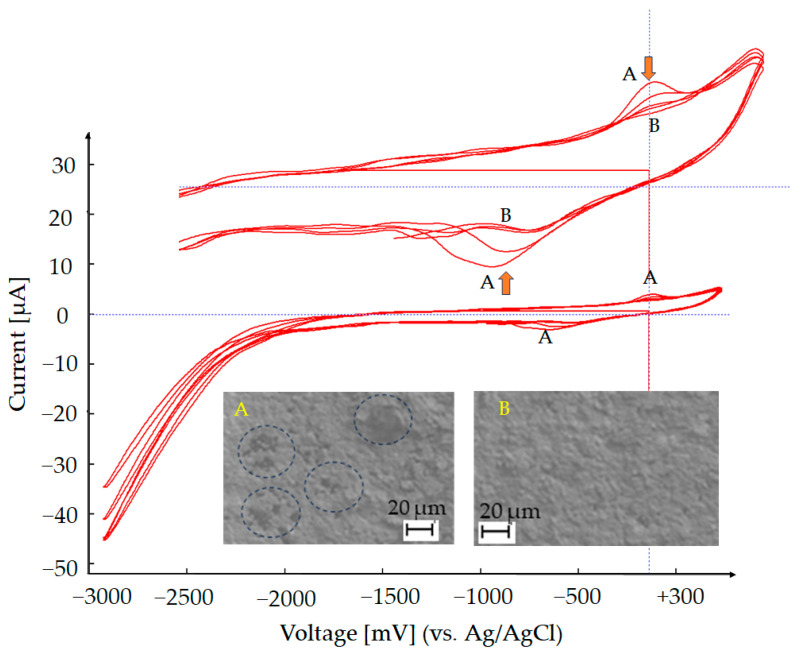
Au deposition via cyclic voltammetry (CV): Au was electrochemically deposited by cycling the potential between −3000 mV and +300 mV at a scan rate of 50 mV/s, using an 18-karat Au plating solution (TIFOO)—Region B. The A/B labels (yellow) are used to indicate specific regions in the SEM image and their corresponding positions in the CV. In this case, region A in the SEM figure highlights an area where the chemical gold coating is not uniform, while region B shows the same area after additional gold electrodeposition.

If the chemically deposited Au layer on the underlying silver electrode is uniform and continuous, the voltammogram shows a clear and stable redox response with minimal background interference. In contrast, an incomplete or porous Au coating results in the appearance of additional peaks associated with the oxidation and reduction of exposed silver. These silver-related signals indicate insufficient Au coverage, allowing electrochemical activity from the silver substrate to contribute to the response. If a silver peak appears during the CV, additional gold can be deposited in situ onto the working electrode using a commercial 18-karat Au plating solution (TIFOO). The electrode is cycled between −3000 mV and +300 mV at a scan rate of 50 mV/s until the silver peak disappears. As shown in Figure 5 the initial scans (Region A) reveal a silver oxidation peak, also confirmed by the SEM image, where the blue circles highlight areas not yet fully covered by gold during the chemical deposition process (A). Following gold deposition, the silver signal gradually diminishes and disappears; in contrast, the gold redox features become more pronounced (Region B), as further confirmed by the SEM image (B), which shows complete coverage of the substrate with gold. This transition confirms the successful formation of a compact Au layer, effectively masking the underlying silver and ensuring a stable and reproducible electrochemical response for subsequent analyses.

The area of the Au electrodes was analyzed using light reflection captured by a camera system in combination with 3D recognition software (ZEUS—developed by Attophotonics). The cut profile beneath the 3D scan shows a square working electrode with a side length of 1.3 mm. The analysis demonstrated a dimensional accuracy of 98.87% for the fabricated electrodes.

### 3.3. Comparative Study of Nickel Electrochemistry in Different Electrochemical Systems

In this chapter, we compare the electrochemical behaviour of nickel ions in two systems: a 0.1 M Na_2_SO_3_ aqueous solution, representative of the high nickel concentrations typically found in industrial settings, and a ChCl–EG deep eutectic solvent designed for lower concentrations. Both systems were studied using our newly developed screen-printed gold electrode array. Table 1 presents the results obtained, benchmarked against state-of-the-art nickel sensors reported in the literature.

This work builds on the current state of the art, highlighting the unique advantages of gold electrodes—their chemical inertness, high conductivity, and compatibility with a wide range of electrolytes and surface modifications. Studies have shown that nickel electrochemistry on gold surfaces is highly dependent on the electrolyte composition, with various strategies, such as complexing agents, redox mediators, and deep eutectic solvents, employed to enhance sensitivity and minimize interference. In our experiments, the relatively high nickel concentrations were intended to simulate industrial environments, where nickel levels commonly range from 10 mM to 500 mM. Our comparative analysis contributes to this field by demonstrating the viability of screen-printed gold arrays for nickel detection in highly concentrated industrial environments and low-concentration media.

This dual-system comparison further underscores the developed platform’s practical potential. It can operate reliably under harsh, industrially relevant conditions where conventional sensors may fail.

Among electrochemical approaches, voltammetry stands out for its simplicity, speed, and ability to detect Ni(II) ions at ultra-trace levels (see Table 1). Modified electrodes—incorporating nanomaterials, polymers, or metal nanoparticles—are commonly used to enhance performance, improving sensitivity, selectivity, and detection limits in environmental monitoring applications [10].

#### 3.3.1. Nickel Electrochemistry in 0.1 M Na_2_SO_3_ System

Nickel concentrations of 48 mM (2.82 g/L) and 91 mM (5.34 g/L) were selected, based on preliminary optimization studies, to enhance electrochemical signal intensity while maintaining solution stability in the Na_2_SO_3_ medium. These concentrations reflect realistic industrial scenarios, and are particularly relevant for sensor applications in nickel sealing and plating baths, where Ni^2+^ levels often exceed 50 mM.

Testing at these levels is critical for evaluating sensor robustness under practical conditions, including industrial process streams and wastewater discharges. By simulating the elevated nickel content typical of such environments, the selected concentrations enable meaningful assessment of the sensor’s performance for both in-process monitoring and environmental compliance.

**Figure 6:**
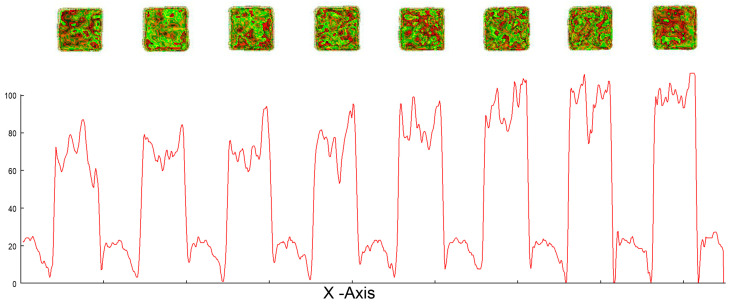
The area of the Au electrodes was analyzed using light reflection captured by a camera system in combination with 3D recognition software (ZEUS—developed by Attophotonics). The cut profile beneath the 3D scan shows a square working electrode with a side length of 1.3 mm. The analysis demonstrates a dimensional accuracy of 98.87% for the fabricated electrodes.

To evaluate the electrochemical performance of the screen-printed electrode array in Na_2_SO_3_ electrolyte, cyclic voltammetry (CV) measurements were conducted, as shown in Figure 6 As the nickel concentration increased, the cathodic current at −1000 mV shifted further in the negative direction (indicated by the blue arrow), while the anodic current also increased (red arrow), suggesting enhanced redox activity due to higher Ni loading on the electrode surface. A cycling protocol was introduced to improve the electrochemical response and better capture concentration-dependent behaviour.

**Figure 7:**
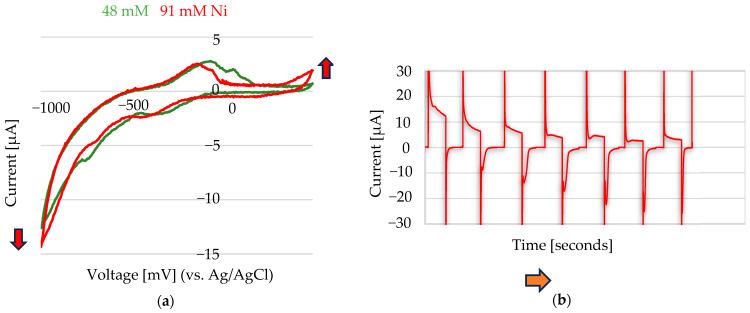
(**a**) Cyclic voltammetry performed at a scan rate of 50 mV/s in 0.1 M Na_2_SO_3_, with added Ni^2+^ from a 1 M NiSO_4_·6H_2_O stock solution: 10 µL (48 mM Ni^2+^) and 20 µL (91 mM Ni^2+^) added to 200 µL of electrolyte. (**b**) Chronoamperometric cycles applied at −1000 mV for 120 ms and 0 mV for 120 ms in 100 µL of 0.1 M Na_2_SO_3_, with increasing volumes of Ni^2+^ stock solution added: 0 µL, 10 µL, 20 µL, 30 µL, 40 µL, 50 µL, and 60 µL, corresponding to final Ni^2+^ concentrations of 0 mM, 91 mM, 167 mM, 231 mM, 286 mM, 333 mM, and 375 mM, respectively. The arrows indicate the direction of increasing nickel concentration. The reference electrode used was Ag/AgCl, and the counter electrode was Au.

**Figure 8:**
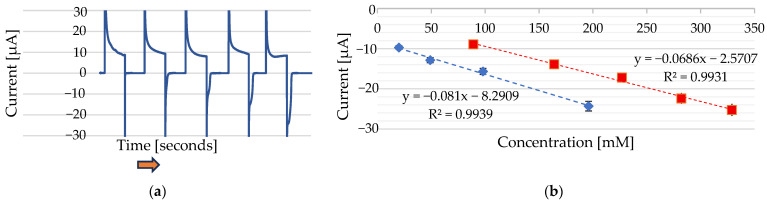
(**a**) Chronoamperometric cycles were applied at −1000 mV for 120 ms and 0 mV for 120 ms in 100 µL of 0.1 M Na_2_SO_3_, with incremental additions of Ni^2+^ from a 1 M NiCl_2_·6H_2_O stock solution. Added volumes of 0 µL, 2 µL, 5 µL, 10 µL, and 20 µL correspond to final concentrations of 0 mM, 20 mM, 48 mM, 91 mM, and 167 mM Ni^2+^, respectively. The reference electrode was Ag/AgCl, and the counter electrode was Au (Au). The arrows indicate the direction of increasing nickel concentration. (**b**) Calibration curves showing the peak current versus the Ni^2+^ concentration using two different stock solutions: 1 M NiCl_2_·6H_2_O (blue line) and 1 M NiSO_4_·6H_2_O (red line).

The Ni^2+^ electrochemistry was studied by cycling at a constant potential of −1000 mV for 120 s, followed by a return to 0 mV for an equal duration of 120 s. This experiment was initially conducted in the absence of Ni^2+^, using only 0.1 M Na_2_SO_3_ (100 µL) as the electrolyte. Incremental additions (2–50 µL) of a 1 M Ni(II) stock solution, prepared from NiSO_4_·6H_2_O (Figure 7b) and NiCl_2_·6H_2_O (Figure 8a), were then added to the electrode containing 0.1 M Na_2_SO_3_ (100 µL).

A linear dependence was observed between the cathodic peak current and the Ni^2+^ concentration when analyzing four Ni^2+^ concentrations ranging from 20 mM to 167 mM (NiCl₂·6H₂O) and when analyzing five Ni^2+^ concentrations ranging from 91 mM to 333 mM (NiSO_4_ 6H_2_O) (Figure 7b). The presence of two linear ranges is attributed to the different electrochemical behaviours of Ni^2+^ ions at the Au electrode when derived from two distinct nickel salts—namely, NiCl_2_·6H_2_O and NiSO_4_·6H_2_O—under identical supporting-electrolyte conditions. Although Na_2_SO_3_ was used in both cases, the accompanying anions (Cl^−^ or SO_4_^2−^) from the nickel salts may still have influenced the electrochemical response of the nickel at the gold electrode, potentially through complexation equilibria or ion-pairing effects. Similar behaviour has been reported for DMG-based sensors [10].

The linear detection ranges were established by measuring the current response across a series of Ni^2+^ standard solutions of known concentrations. The linear ranges were calculated by plotting the peak current against the corresponding Ni^2+^ concentrations and applying linear regression with a 95% prediction interval. The range within the current response that remained proportional to concentration was defined as the linear range.

The analytical performance of the SPAu electrode was evaluated for Ni^2+^ detection in test samples under optimum conditions. The limit of detection (LOD) of the Ni^2+^ was determined according to the following equation:

LOD = 3σ/slope

where σ represents the standard deviation of the blank signal, and the slope corresponds to the gradient of the calibration curve. Based on this method, the LOD was determined to be 27.35 mM for Ni^2+^ from NiSO_4_·6H_2_O and 0.79 mM for Ni^2+^ from NiCl_2_·6H_2_O.

#### 3.3.2. Nickel Electrochemistry in ChCl-EG System

The study of metal ion speciation in deep eutectic solvents (DESs) is more complex than that in aqueous solutions, due to the varying Lewis basicities of the anions and the specific eutectic composition. Understanding speciation is essential for unravelling the mechanisms of nucleation and growth in metallic coatings, as ligand binding constants dictate the steps of electrochemical reduction. However, progress is hindered by the difficulty in obtaining quantitative structural data, as most metal complexes in DESs cannot be crystallized or isolated by solvent evaporation. Smith et al. demonstrate that due to the high Cl^−^ concentration from ChCl, [NiCl_4_]^2−^ is the dominant complex in DESs. In the EG-only system, where Cl^−^ comes from added metal halides and electrolytes, the lower Cl^−^ concentration makes [NiCl_3_(EG)_3_]^−^ the more favourable complex [51]. The electrodeposition process is complex, and there are multiple parallel reactions, which may include different contributions, such as adsorption (also related to the self-limiting growth of nanoclusters), proton reduction, water reduction (if used NiCl_2_x6H_2_O as Ni ions sources), etc. Cui et al. show that in this system, nickel reactions follow a three-dimensional transient nucleation mechanism encompassing three processes: adsorption, diffusion-controlled three-dimensional nucleation/growth, and water reduction [50]. Water reduction in deep eutectic solvents (DESs) typically occurs at more negative potentials, around −1.0 to −1.2 V, versus Ag/AgCl. However, suppose water is present as a trace impurity. In that case, it might begin reducing at a potential close to that of nickel reduction, potentially leading to hydrogen evolution, which can compete with nickel deposition at higher applied potentials. In this study, water-free NiCl_2_ was used. Although the fundamental relationship I = Q/t (current equals charge over time) applies to nickel deposition from DESs, several system-specific factors must be considered to interpret deposition behaviour accurately. These include the electrolyte composition, current efficiency, overpotentials, and overall conductivity of the solution. DESs typically exhibit lower conductivity than aqueous electrolytes, which results in lower achievable current densities under the same applied voltage. Consequently, to maintain comparable nickel deposition rates, the system may require higher voltages, elevated temperatures, increased salt concentrations, or optimized plating conditions to enhance mass transport and charge transfer.

**Figure 9:**
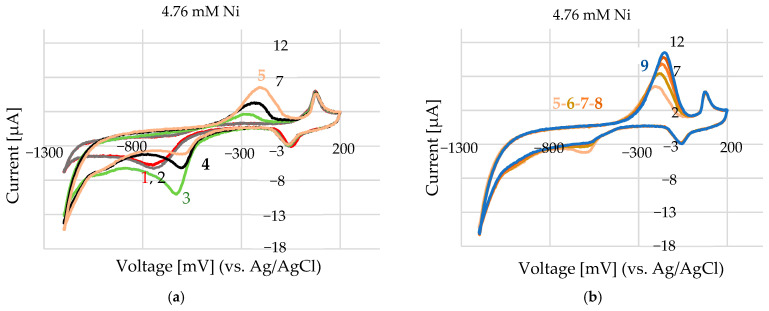
Cyclic voltammetry of 4.76 mM Ni^2+^ in a deep eutectic solvent (100 mM choline chloride in ethylene glycol): CV was performed with a potential range from +200 mV to −1200 mV at a scan rate of 50 mV/s. (**a**) Initial scans (1–5) showing the accumulation behaviour of nickel at the electrode surface. (**b**) Final scans (scan numbers 5 to 9, no. 9 shown in blue) after repeated cycling, highlighting changes in the current response due to electrode surface modification or nickel deposition.

Measurements were made at room temperature in air and under argon gas, and produced identical CV scans. The CV measurements were performed as it follows: first, the electrodes were submerged in the supporting solution, and a scan was performed to obtain a background measurement. Then, the working electrode, which was already covered with the supporting solution (100–200 µL), was submerged in nickel-containing solution (in µL range), and the scan was started. The study of 4.76 mM Ni in 100 mM ChCl in EG (Figure 8a) shows that at the beginning (grey and red line), an adsorption process takes place (−750 mV and −6.2 µA–3.6 µA/mm^2^). Significant energy is needed to form nuclei by gathering atoms in the system, as close proximity between atoms can facilitate the formation of larger atomic nuclei on clusters. In systems with significant internal resistance—particularly those involving concentrated electrolytes or poorly conductive solvents—an increase in current can lead to a shift in the measured potential toward more positive values (Figure 9a—green line). This phenomenon, known as IR drop (voltage drop due to internal resistance), becomes especially pronounced in low-conductivity environments such as certain deep eutectic solvents (DESs). In these systems, high currents can amplify resistance-related distortions, affecting the accuracy of potential measurements and the interpretation of electrochemical responses. In diffusion-limited systems, where the supply of reactants to the electrode surface cannot keep up with the electron-transfer rate, increasing the current can push the system toward a diffusion-limited state. In such cases, the potential might shift in the positive direction as the system struggles to sustain the desired reaction at a higher current. The deposited layer’s formation influences the oxidation peak, and its appearance depends on the kinetics of the nucleation and growth process. Therefore, the oxidation peak does not necessarily appear immediately, but rather after sufficient nucleation, when there is enough deposited material for a detectable oxidation current (Figure 9a)—noted with no.5, initial stage and continuation in (Figure 9b) until the system is stable. At the positive limit, the electrochemical stability of the solvent is constrained by the oxidation of chloride anions into chlorine, which is solubilized as Cl_3_^−^ rather than evolving as gaseous Cl_2_. Depending on the electrode material, such as Au, oxidation can occur at less positive potentials, leading to the formation of a soluble Au chloride complex, [AuCl_2_]^−^, due to the high chloride concentration in the DES, thereby explaining the relatively narrow potential window of this material.

T. Doneux et al. [52] demonstrated that the voltammetric characteristics of silver electrodeposition and stripping on Au working electrodes in various DESs were comparable in both the overpotential and underpotential range as a result of identical Ag(I) speciation controlled by the high chloride concentrations in these media. Cathodic and anodic peaks were observed between −100 mV and +100 mV [52]. In the present system, the peaks at +80 mV and −60 mV are attributed to the Ag/AgCl redox on the Au electrode in this media.

A concentration of 4.76 mM Ni^2+^ was selected as a representative value for evaluating the electrochemical performance of the screen-printed electrode array. Positioned at the lower end of concentrations commonly found in industrial processes such as nickel sealing baths (typically ~50 mM), it provides a practical balance—low enough to avoid complications like high current densities or electrode fouling, yet high enough to yield stable, reproducible signals. Moreover, this concentration lies within the sensor’s linear detection range, making it well-suited for calibration, repeatability testing, and comparisons with real industrial samples. The experiment illustrated in Figure 9a was conducted using 4.76 mM Ni^2+^, capturing the initial scans (1 to 5). Subsequent scans (5 to 9) are shown in Figure 9b, with scan 9 highlighted in blue.

**Figure 10:**
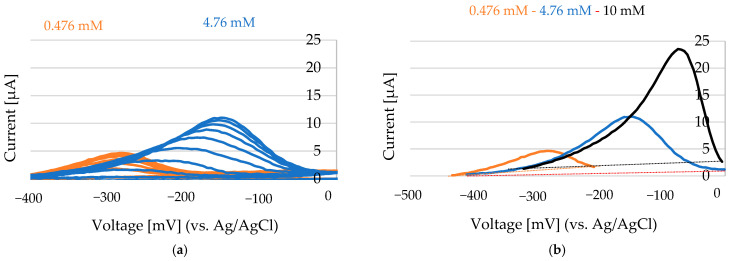
NiCl_2_ concentrations of 0.5 mM, 4.8 mM, and 10 mM (in 100 mM Choline Chloride in Ethylene glycol). Scans conducted at 200 mV to −1200 mV at a rate of 50 mV/s. (**a**) Selected anodic peaks between −400 mV and 0 mV. After the addition of NiCl_2_ (5 µL of 100 mM NiCl_2_ on the Au electrode that was covered with 100 µL of 100 mM ChCl-EG), the anodic peak increases continuously. (**b**) The last scans after anodic current stabilization for 0.475 mM Ni, 4.76 mM Ni, and 10 mM Ni.

For nickel determination in this system using a Au working electrode, a Au counter electrode, and a Ag/AgCl reference electrode, both the anodic and cathodic currents can serve as indicators of Ni^2+^ concentration. As shown in Figure 10a (before stabilization) and Figure 10b (after stabilization), the anodic current—measured after the system reaches electrochemical equilibrium—is generally more reliable. This anodic response reflects the amount of nickel previously deposited during the cathodic phase and offers greater stability and reproducibility for quantification. In contrast, the initial cathodic current may also correlate with nickel concentration, but its accuracy is limited by factors such as double-layer charging and nucleation effects, which introduce variability. Due to these dynamic influences at the onset of deposition, the cathodic signal is often less consistent. Therefore, the anodic current after stabilization is typically the preferred measurement parameter for accurate and stable nickel quantification.

**Figure 11:**
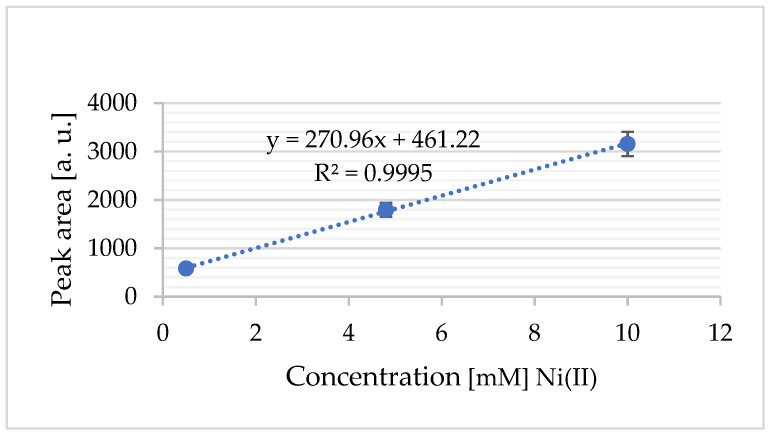
The standard addition calibration plot for Ni^2+^ (from NiCl_2_): a calibration curve generated using the standard addition method, illustrating the linear relationship between the added Ni^2+^ concentration and the corresponding electrochemical response (current signal).

By measuring the area under the anodic peak and integrating this value into a standard addition plot, the concentration of oxidized nickel was determined (Figure 11). The standard deviation was calculated from n = 3 independent replicates.

**Figure 12:**
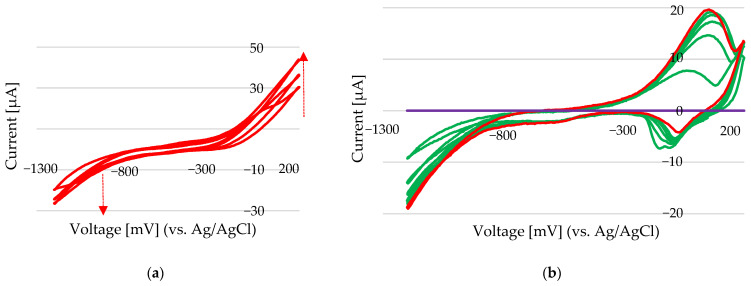
(**a**) CV scans at a rate of 50 mV/s for 10 µL of 100 mM NiCl_2_ in 100 µL of 100 mM ChCl-EG (9 mM Ni). Background: purple line—the new Au electrode in 100 mM ChCl-EG, green line—the last 5 scans, and red line—the CV scan of 9 mM Ni after stabilization. (**b**) CV scans at a rate of 50 mV/s for 10 µL of 100 mM NiCl_2_ in 100 µL of 100 mM ChCl-EG (9 mM Ni) after 30 s of deposition at −1200 mV.

When nickel was pre-deposited—for instance, by applying −1200 mV for 30 s—and a subsequent cyclic voltammetry scan was performed, noticeable shifts in the current peaks in both the anodic and cathodic regions were observed (Figure 12a). However, no distinct oxidation peaks appeared during these scans. This behaviour may be attributed to surface interactions within the deep eutectic solvent (DES), specifically choline chloride–ethylene glycol (ChCl-EG). In this chloride-rich environment, nickel may form surface-stabilized complexes such as NiCl_x_ (x = 1, 2), which can contribute to surface passivation.

The formation of a thin nickel chloride layer on the electrode surface might have suppressed oxidation processes and altered the electrochemical response. Energy-dispersive X-ray spectroscopy (n) analysis (Au electrode) suggested that the deposited layer had a nickel-to-chloride atomic ratio consistent with that of nickel chloride, supporting the hypothesis of surface adsorption or in situ formation of Ni–Cl complexes during electrochemical cycling. In DES systems, where chloride ions are abundant and solvent interactions are complex, these surface-bound species likely play a significant role in modifying electron-transfer kinetics. Under these conditions, standard addition experiments performed over the potential range of −1300 mV to +200 mV showed that the anodic peak, after system stabilization, provides a reliable signal for Ni^2+^ quantification (Figure 12b). This anodic current reflects the amount of nickel deposited and is not significantly affected by interfering oxidation reactions, making it suitable for analytical determination.

During a potential scan from 0 mV to −1300 mV at a scan rate of 50 mV/s (Figure 12), nickel deposition commenced with an initial cathodic current of −19 µA. The cathodic peak progressively decreased with continued scanning, reaching −3.4 µA by the final scan. Following deposition, the first oxidation peak appeared at approximately +3.4 µA. It increased steadily to 13.7 µA in subsequent cycles, indicating enhanced oxidation activity as more nickel was deposited on the electrode surface.

To estimate the absolute amount of nickel deposited, Faraday’s law of electrolysis was applied using the following equation:

$$m=\frac{M\;I\;t}{nF}$$

*M* = 58.69 g/(molar mass of nickel), *I* = current (A), *n* = 2n (number of electrons for Ni^2+^ reduction), and *F* = 964.85 C/mol (Faraday’s constant). The example below considers a deposition time of *t* = 240 s.

**Figure 13:**
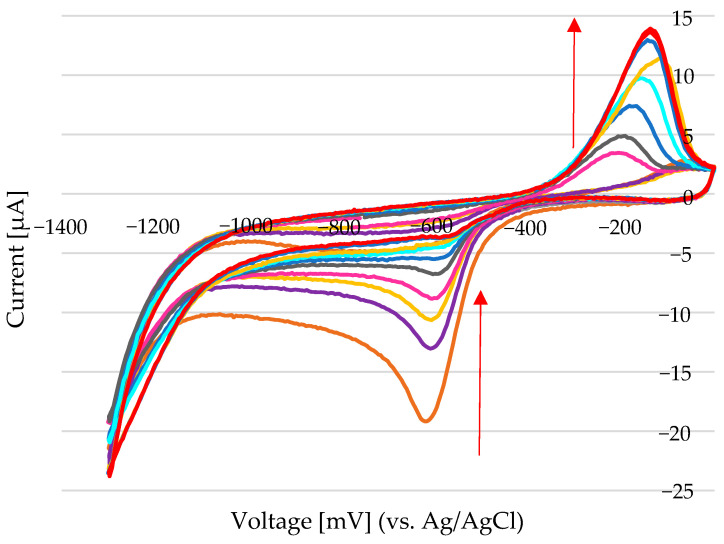
Cyclic voltammetry was performed in a solution containing 1.9 mM Ni^2+^ (from NiCl_2_) dissolved in 100 mM choline chloride in ethylene glycol (ChCl–EG). The potential was scanned from 0 mV to −1300 mV at a scan rate of 50 mV/s. Nickel deposition was initiated at a cathodic current of −19 µA, with the peak current gradually decreasing to −3.4 µA over subsequent scans. The first oxidation peak appeared at +3.4 µA and increased steadily, reaching 13.7 µA, indicating progressive nickel accumulation and enhanced anodic activity with continued cycling. The arrow indicates the decrease of the reduction peak and the increase of the oxidation peak, from the first scan to the last scan.

For example, with a cathodic current of −19 µA (equivalent to −11.18 µA/mm^2^ for the given electrode area) and a nickel concentration of 1.9 mM (Figure 13), assuming a deposition time of 240 s, the calculated amount of deposited nickel is approximately 1.51 nanomoles (nM).

However, it is important to note that while the initial cathodic current may correlate with the nickel ion concentration, it is not always a reliable metric, due to transient effects such as double-layer charging and nucleation phenomena. These effects can vary between scans and introduce inconsistencies, especially during the early stages of metal deposition. Therefore, it is often preferable to rely on stabilized anodic signals post-deposition for accurate quantification.

The electrochemical behaviour of Ni^2+^ in choline chloride–ethylene glycol (ChCl–EG) was studied using the novel screen-printed Au electrode array. Under optimized experimental conditions, a strong linear relationship (R^2^ = 0.9995) was observed between the peak current and the Ni^2+^ concentration in the range of 0.5 mM to 10 mM, with a calculated detection limit of 1.6 µM, demonstrating the system’s suitability for quantitative electrochemical analysis in DES-based media.

**Figure 14:**
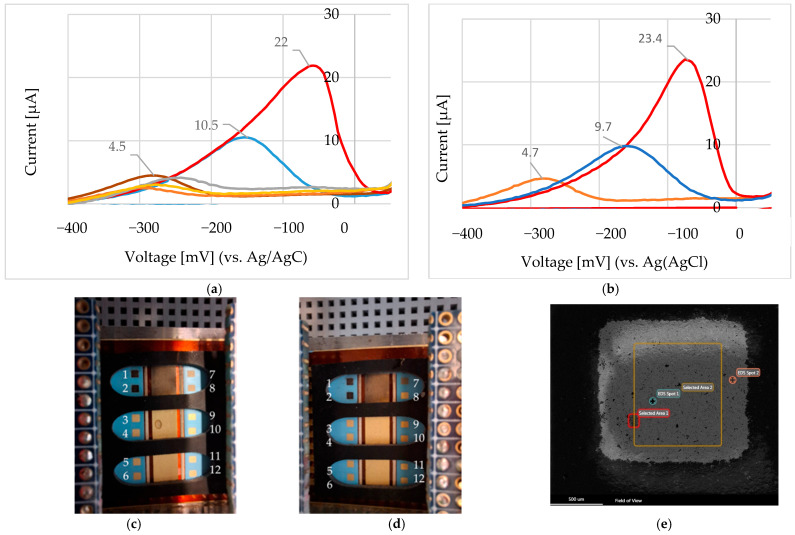
(**a**) Cyclic voltammetry (CV) responses for 0.476 mM (electrode 3 in panel c), 4.76 mM (electrode 4), and 10 mM (electrode 5) Ni^2+^, using NiCl_2_ in 100 mM ChCl–EG. Potential range: +200 mV to −1200 mV; scan rate: 50 mV/s. (**b**) Identical experiments performed on electrodes 2, 4, and 5 from the array shown in panel (**d**). (**c**) Electrode array (left side, L), labelled E1–E5; (**d**) right-side array (R), labelled E1–E5. Electrodes 1, 2, 7, and 8 in panels (**c**,**d**) were used for tests in 0.1 M Na_2_SO_3_. Dark areas indicate deposited Ni species (oxides/hydroxides). (**e**) SEM image of electrode E1-L: 4 wt% Ni in Area 2; 1.2 wt% Ni in Spot 2.

We applied the same electrochemical procedure across multiple electrodes within the screen-printed gold array to assess reproducibility. Figure 14a,b show the cyclic voltammetry (CV) results for 0.476 mM, 4.76 mM, and 10 mM Ni^2+^, as described in Figure 9b. These measurements were conducted on electrodes 3, 4, and 5 from the left-hand side of the array (Figure 14c), and electrodes 3, 4, and 5 from the right-hand side (Figure 14d), respectively. In all cases, NiCl_2_ was dissolved in 100 mM choline chloride–ethylene glycol (ChCl–EG), and the potential was scanned from +200 mV to −1200 mV at 50 mV/s. The photographs in Figure 14c,d show representative electrode surfaces after electrochemical testing, with visible darkened areas indicating the formation of Ni-containing species, such as nickel oxides and hydroxides. SEM characterization of electrode E1-L (Figure 14e) confirmed these deposits, revealing 4 wt% Ni in Area 2 and 1.2 wt% in Spot 2. Electrodes 1, 2, 7, and 8 were reserved for experiments in a 0.1 M Na_2_SO_3_ electrolyte system.

**Figure 15:**
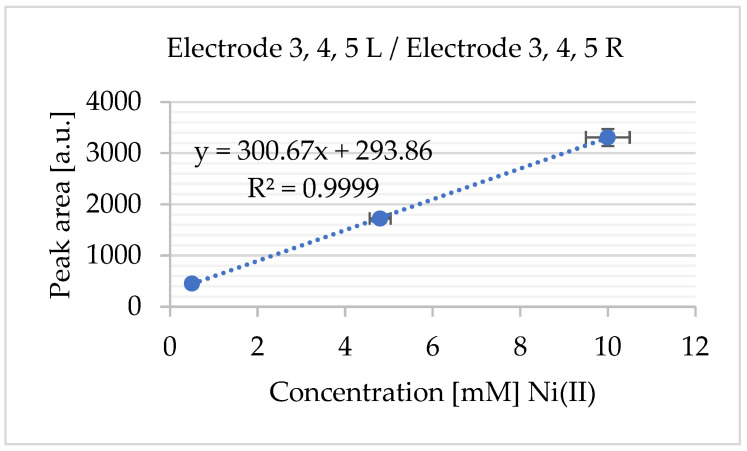
Standard addition calibration plot for Ni^2+^ (from NiCl_2_): The calibration curve constructed using the standard addition method shows the linear relationship between the added Ni^2+^ concentration and the corresponding electrochemical response (current signal). The data were derived from the experiments in Figure 14a,b. The standard deviation was calculated from n = 3 independent replicates.

Although Figure 14c,d primarily serve as visual references for assessing electrode integrity following electrochemical testing, the corresponding CV results (Figure 14a,b), together with the standard addition calibration plot for Ni^2+^ (Figure 15), demonstrate consistent electrochemical responses across multiple electrodes in the array. These results underscore the reproducibility of the fabrication process and confirm the array’s potential for reliable multiplexed sensing applications.

The electrochemical behaviour of Ni^2+^ in choline chloride–ethylene glycol (ChCl–EG) was studied using a novel screen-printed Au electrode array. Under optimized experimental conditions, a strong linear relationship (R^2^ = 0.9995) was observed between the peak current and the Ni^2+^ concentration in the range of 0.5 mM to 10 mM, with a calculated detection limit of 1.6 µM, demonstrating the system’s suitability for quantitative electrochemical analysis in DES-based media.

## 4. Conclusions

A screen-printed sensor system was developed, featuring a 16-electrode array coated with a thin gold layer deposited via a chemical method, which can be further enhanced through electrochemical gold deposition. This design enables high customisability and supports subsequent functionalisation strategies. The key advantage of this platform lies in its flexibility and potential for simultaneous multi-analyte detection, which can be achieved through selective functionalisation of individual electrodes within the array.

The electrochemical behaviour of Ni^2+^ was investigated using the screen-printed gold electrode array in two distinct electrolyte systems: aqueous Na_2_SO_3_ and a deep eutectic solvent (DES) composed of choline chloride and ethylene glycol (ChCl–EG). Incremental additions of Ni^2+^ (2–50 µL) were introduced using NiCl_2_·6H_2_O and NiSO_4_·6H_2_O in Na_2_SO_3_ and anhydrous NiCl_2_ in ChCl–EG. The current investigation aimed to demonstrate the feasibility of fabricating and applying novel screen-printed electrode arrays for Ni^2+^ electrochemistry, particularly in contexts relevant to industrial environments, where nickel concentrations are significantly higher (typically 10–500 mM).

The sensor’s analytical performance was evaluated across both electrolyte systems. In Na_2_SO_3_, linear detection ranges were observed between 20 and 196 mM for NiCl_2_·6H_2_O, and between 89 and 329 mM for NiSO_4_·6H_2_O, with corresponding limits of detection (LODs) of 0.79 mM and 27.35 mM. In the ChCl–EG system, the sensor exhibited a linear range between 0.5 and 10 mM (R^2^ = 0.9995) and an LOD of 1.6 µM, confirming its suitability for lower-concentration applications.

These results indicate that while the Na_2_SO_3_-based measurements are suited for higher concentration applications, the ChCl–EG system offers promising potential for further refinement toward trace-level detection. Future work will focus on optimizing the electrode surface modification and detection protocols to enhance sensitivity and bring the detection limits closer to regulatory requirements.

The array design allows individual electrodes to be selectively modified with chemical recognition elements, such as ligands, ion-selective membranes, or enzyme coatings. These can be applied using localized techniques like inkjet deposition. For example, certain electrodes could be functionalised with nanomaterials or thiol-based capture molecules to target specific metal ions or molecular analytes. This modular approach supports parallel analysis and signal deconvolution strategies, enabling real-time, multiplexed sensing within a single measurement cycle. Further exploration of this functionalisation concept is planned to demonstrate the full multiplexing capabilities of the array.

Figure 13 and Figure 14 demonstrate consistent electrochemical responses across different electrodes, highlighting the reproducibility of the fabrication process. This reproducibility is critical for sensor platforms intended for multi-analyte or high-throughput applications. The potential interference from coexisting metal ions will be explored in future investigations, with particular emphasis on assessing the sensor’s selectivity and performance in complex, real-world matrices. Analyzing real samples is important for validating sensor performance under practical conditions. A forthcoming study, which will include environmental and industrial samples to assess their reliability and selectivity in complex systems, plans to apply these electrodes to real sample matrices.

While the present study focused on the fabrication and electrochemical evaluation of flexible screen-printed gold electrode arrays for nickel(II) sensing, we acknowledge the importance of long-term stability assessments, and have identified this as a critical direction for future research to support the further development and real-world applicability of these sensor platforms.

Additionally, the platform’s modular design opens the door to multiplexed sensing applications targeting a broader range of environmentally and biologically relevant analytes, including other heavy metals, nitrates, and pesticides. Through surface modification with thiol–Au chemistry and molecular recognition elements, the system could also be adapted for biosensing in food safety and medical diagnostics.

## Figures and Tables

**Table 1:** An overview of representative sensors previously reported for the voltammetric detection of Ni^2+^ ions. * Converted to µg·L^−1^ based on reported data for unit consistency.

Substrate	Technique	Accumulation Time (s)	Dynamic Linear Range (µgL^−1^)	Detection Limit (µgL^−1^)	Reference
AgNPs/Fe_3_O_4_/DMG/CPE	SWSV	60	0.002–0.01 µM	0.0006 µM	[11]
			0.12–0.59 *	0.035 *	
ICP-MS				0.1	
PbF-SPE	SWAdS	60	0.6–2.9	0.20	[12]
Fe_3_O_4_@MWCNTs@IIP	DPASV		0.5–20 and 20–200	0.27	[13]
RBiABE	DP AdSV	30	0.59–41	0.29	[14]
AAS				0.5	
Carbon-SPE	DPAdSV	120	1.7–150	0.5	[15]
Pt/Mn_3_O_4_-Chitosan	CV	18	5–250	0.718	[16]
MWCNT-BiOCl nanosheets	Chrono deposition	60	20–160 µM	0.019 µM	[17]
	with DPV stripping		1174–9390 *	1.115 *	
NGr-DMG-GCE	AdCSV	120	2–20	1.5	[18]
Carbon-SPE	AdSV	60	7.6 to 200	2.3	[19]
Bi-Graphene-SPE	SWAdS	120	10–40	2.5	[20]
Ag/BiOBr	CV	-	3.4–13.6 µM	0.122 µM	[21]
			199.5–798.2 *	7.2 *	
ICP-AES				10	
Bismuth biomaterials	CV, LSV	--	1.7–170 µM	0.477–1.669 µM	[22]
			99.7–9977 *	28–97.9 *	
Au-SPE -calixarene	DPV	120	340	130–1680	[23]
Pt/Pt	CV	-	15–40 mM 880,350–2347,600 *	274.99 ppm 274,990 *	[24]
Au-SPE-NiCl_2_ 6H_2_O-NaSO_3_			20–196 mM ** 119.7–1380.38 µg absolute value	0.79 mM ** 4.74 µg absolute value	This work
Au-SPE-NiSO_4_ 6H_2_O-NaSO_3_			89–329 mM ** 574.58–2896.35 µg	27.35 mM ** 176.6 µg absolute value	This work
Au-SPE-NiCl_2_-ChCl-EG	CV	-	0.5–10 mM ** 2.9–61.6 µg absolute value	1.6 µM ** 0.72 µg absolute value	This work

** Under the assumption of 100% faradaic efficiency over 240 s, it is theoretically possible to deposit up to 0.79 µg of Ni from a 0.476 mM solution, 1.23 µg from 47.6 mM, 1.44 µg from 9 mM, and 1.76 µg from 10 mM on a 1.7 mm^2^ working electrode. These values correspond to current densities ranging from −6.81 to −13.35 µA/mm^2^. Given that the electrolyte volume is limited to 105 µL, the total amount of Ni^2+^ available in the solution at the electrode is 2.9 µg (0.476 mM), 55.5 µg (9 mM), and 61.6 µg (10 mM). While these are theoretical estimates, they suggest that the system operates well within a quantifiable range between the limit of detection (1.6 µM) and the tested maximum of 10 mM. For higher concentrations, the electrolyte volume varied between 120 µL and 150 µL.

## Data Availability

Data are contained within the article.

## Abbreviations

The following abbreviations are used in this manuscript:

AAS	Atomic Absorption Spectroscopy
AdCSV	Adsorptive Cathodic Stripping Voltammetry
AdSV	Adsorptive Stripping Voltammetry
AgNPs	Silver Nanoparticles
Calix/MPA/Au	Calix[4]arene–Mercaptopropionic Acid Modified Au Electrode
ChCl-EG	Choline Chloride–Ethylene Glycol (Deep Eutectic Solvent)
CV	Cyclic Voltammetry
DES	Deep Eutectic Solvent
DMG	Dimethylglyoxime
DP AdSV	Differential Pulse Adsorptive Stripping Voltammetry
DPASV	Differential Pulse Anodic Stripping Voltammetry
DPV	Differential Pulse Voltammetry
EDTA	Ethylenediaminetetraacetic Acid
EDX	Energy-Dispersive X-ray Spectroscopy
EG	Ethylene Glycol
GDC	Glassy Carbon Electrode
HER	Hydrogen Evolution Reaction
ICP-AES	Inductively Coupled Plasma—Atomic Emission Spectroscopy
ICP-MS	Inductively Coupled Plasma—Mass Spectrometry
LSV	Linear Sweep Voltammetry
OER	Oxygen Evolution Reaction
PbF-SPE	Plated Lead-Film Screen-Printed Electrode (PbF-SPE)
RBiABE	Bismuth Bulk Annular Band Working Electrode
SEM	Scanning Electron Microscopy
SMAMQ	Sulfamethoxazole-quinolinol
SPAu	Screen-Printed Au Electrode
SWAdS	Square Wave Adsorptive Stripping Voltammetry
SWASV	Square Wave Anodic Stripping Voltammetry
SWSV	Square Wave Stripping Voltammetry
USEPA	United States Environmental Protection Agency
WHO	World Health Organization
XPS	X-ray Photoelectron Spectroscopy
